# Measuring the Capacity Utilization of Public District Hospitals in Tunisia: Using Dual Data Envelopment Analysis Approach

**DOI:** 10.15171/ijhpm.2016.66

**Published:** 2016-06-06

**Authors:** Chokri Arfa, Hervé Leleu, Mohamed Goaïed, Cornelis van Mosseveld

**Affiliations:** ^1^Iational Institute of Labour and Social Studies (INTES), University of Carthage, Tunisia, Tunis.; ^2^LEM-CNRS, IÉSEG School of Management, Lille, France.; ^3^LEFA-IHEC, University of Carthage, Tunisia, Tunis.; ^4^Health Economist Expert (free lance).

**Keywords:** Data Envelopment Analysis (DEA), Shadow Prices, Capacity Utilization (CU), Public District Hospitals (PDHs), Tunisia

## Abstract

**Background:** Public district hospitals (PDHs) in Tunisia are not operating at full plant capacity and underutilize their operating budget.

**Methods:** Individual PDHs capacity utilization (CU) is measured for 2000 and 2010 using dual data envelopment analysis (DEA) approach with shadow prices input and output restrictions. The CU is estimated for 101 of 105 PDH in 2000 and 94 of 105 PDH in 2010.

**Results:** In average, unused capacity is estimated at 18% in 2010 vs. 13% in 2000. Of PDHs 26% underutilize their operating budget in 2010 vs. 21% in 2000.

**Conclusion:** Inadequate supply, health quality and the lack of operating budget should be tackled to reduce unmet user’s needs and the bypassing of the PDHs and, thus to increase their CU. Social health insurance should be turned into a direct purchaser of curative and preventive care for the PDHs.

## Background

Tunisian health sector reform was officially started in the mid-1990s aiming at improvement of health quality, social health protection and the performance of the health system. The health system is predominantly public as 85% of all beds belong to public hospitals. The medical doctor’s density is inequitably distributed and is lower in poorer areas, where most beneficiaries are covered by the Ministry of Health (MoH) through free medical assistance. The main steward is the MoH although its oversight of the private sector is currently limited.

The public hospitals are relatively well-distributed geographically in terms of physical facilities but remains facing challenges in terms of quality, access and performance. It is organized in three levels: primary level including 2104 primary healthcare centers and 105 Public district hospitals (PDHs), secondary level including 32 regional hospitals to which urban PHC facilities are linked and a third tier composed from 22 university hospitals, which are mainly located in large urban cities. Public hospitals consume 51% the total current health expenditure and 90% of public health funding, of which 22% is dedicated to PDH. Hospital budgets are composed of state subsidies from the annual budget, reimbursement through billing system for patients covered by social health insurance (only for regional and university hospitals) and by user-fee revenue. Capital investments and pay roll staffing are MoH’s responsibility while operating costs are covered under a prospective line-item. In this context, incentives for hospitals to operate efficiently are generally weak.

During the last two decades, Tunisia has implemented reforms of the health sector and of the social health insurance. The financing and managerial autonomy has been increased, more specifically for university and regional public hospitals.^1^ Teaching hospitals have been given varying degrees of semi-autonomy within the public sector and empowered to make key strategic, financial, and clinical decisions themselves. The social health insurance reform has been ongoing into two main objectives. The first is devoted to increase access for both public and private health facilities while the second is dedicated to improve finance and performance of the health system. Social health protection is being provided by the National Health Insurance fund and medical assistance schemes for poor and vulnerable.^1^ Nevertheless, Tunisia’s health system continues facing huge challenges. Healthcare resources are unequally distributed across the country. The country lacks an effective primary care system, represented by basic healthcare centers and district hospitals.

Actually, public hospitals receive 33% of total current health expenditures and 55% of total outpatient and inpatient’s care in the country.^2^ The PDH are receiving meager resources (20%-25% of the total MoH funding^3,4^) with a lack of clear strategic planning and management. Despite the budget constraint, public hospitals are providing 80% of total inpatient’s care.

In Tunisia, hospital capacity was evaluated using input-based indicators (bed occupancy, length of stay, etc) or inpatient days and number of beds. The bed occupancy rate (BOR) is a measure of utilization of the available beds, more precisely the percentage of occupied beds. The PDH experienced low BORs. BORs fluctuated around 25%-38% during a long period, low compared to teaching hospitals (75%-80%), regional hospitals (50%-55%)^3,5^ and to conventional accepted levels (80%-85%).

PDH experienced higher excess capacity to satisfy uncertain demand and consequently higher costs. Excess capacity can be explained by the low rate of the bed occupation that has never been more than 38% and in average 34% in 2000 and 25% in 2013. These hospitals seem treating more outpatient’s people rather than being admitted to these hospitals. There is also very little price competition and little incentive to contain costs and ensure efficiency and plant capacity utilization (CU).

Excess capacity is due to many factors like the inadequate healthcare supply, the lack of budget and the shortage of human resources, particularly specialized physicians and to the uncertain nature of healthcare demand.^6^ PDH are managed at central MoH level in recruitment, procuring of supplies and capital investment decisions. PDH are historically struggling with the lack of internal strategic planning and management for an effective primary healthcare delivery. PDH offer healthcare services not in line with clinical requirements of non-communicable diseases and longterm treatment whereof it does not meet the needs of large proportion of the population.

There is a huge gap on individual public health facility performance due to the lack of research evidence, particularly on efficiency and CU. Inefficiency and low rates of CU weaken the health system’s effectiveness and waste resources. This study develops an economic framework for analyzing the plant CU and optimal input usage on the short term for PDH s using an extended data envelopment analysis (DEA) model. It seeks to examine the CU for a sample of PDH between two years, 2000 and 2010, with a view to assess the regional distribution. It will generate evidence for managers and planners in resource allocation decisions.

The economic and engineering concept of CU according to Johansen^7^ is preferred in the context of multiproduct hospitals. In empirical literature, a range of methods for measuring CU has been developed. The prominent are nonparametric frontier analysis based on linear program DEA^8-10^ approaches for CU estimates.^11-15^ The CU is estimated from data on observed inputs and outputs. Many times the concept of capacity is closely related to the technological characteristics of the production process. For this reason, DEA has the great advantage that it does not require any a priori specification about a particular functional form and this ensures the sufficient flexibility to adapt to the specific characteristics of the observed production unit.

The structure of this paper is as follows. The background was discussed in this first section. The theoretical model (methods) and data are discussed in the second section. The results of the analyses are presented in the third section. The last section includes a discussion of results and the conclusions.

## Methods

### Theoretical Model and Estimation

Theoretical foundation of CU is provided by Johansen^7^ and Morrison.^14^ Johansen referred to the production function and defined single output capacity technology as: *“Production capacity is defined as the maximum that can be produced by a production unit with fixed and variable inputs for a given period and provided that the availability of variable factors of production is not restricted.”*

Nelson^16^ and Morrison^14^ provide the economic definition of capacity, where the optimal output measure is the tangency between the short-run and long-run average cost curve.

Many Studies^12,13,15,17^ have focused on Johansen’s definition assessing individual hospitals CU. CU corresponds to output produced, given full and efficient variable input utilization and capacity base constraints imposed by ie, fixed factors, technology, environmental conditions, and resource stock. Frontier setting preference using distance function^18^ with the DEA approach^10,13^ as key method is reasoned as DEA does not require input price information and can incorporate multiple outputs. DEA is based on Koopmans^19^ and Farrell^20^ (economic axioms). Arrow and Debreu^21^ provides the production technology and frontier enabling the scores estimation of efficiency and CU. In frontier setting, the CU scores can be estimated using the distance function^10,12^ or the directional distance function.^22,23^

Färe^12,13,15^ has developed a primal DEA model estimating CU from output-oriented efficiency scores. DEA constructs a ‘‘best practice frontier’’ for maximum possible outputs for fixed input quantities. DEA has been extended examining sufficient capacity among hospitals and their CU. Following Färe,^11^ we adopt Johansen’s definition of plant CU: *‘‘The maximal amount that can be produced per unit of time with existing plant and equipment without restrictions on the availability of variable production factors.*’’^8^

To estimate individual PDH CU, we formulate a dual DEA model including additional constraints on input and output shadow prices. Shadow price restrictions enrich the empirical CU measure by adding priorities in terms of input and output costs which have a significant economic meaning. It is well-known that DEA models are very flexible in the sense that they evaluate an observation in its best possible light. Therefore, no a priori ordering is made among the shadow prices of inputs or outputs and all positive shadow prices are allowed for the different inputs and outputs. However, in order to maximize the efficiency of the evaluated observation, extreme shadows prices can be selected by the linear program which can be counterintuitive from an economic point of view. We can limit this extreme flexibility of DEA models by constraining the ordering of shadows prices. While not knowing the ‘real’ or ‘market’ prices of each input and output, we can nevertheless order theses prices and impose the same order to the shadow prices. The objective can be thought of as adding an economic meaning into the linear program of DEA.

Summarizing, CU scores are obtained in three steps determining (*a*) the maximum output obtainable from observed (fixed and variable) inputs; (*b*) the maximum amount of output obtained from observed fixed inputs alone assuming unconstrained variable inputs; and (*c*) take the ratio of the first two steps to obtain a CU measure.^9^ Steps (*a*) and (*b*) require a series of the linear programming steps.

Assuming identical hospital production technology, the production technology set *(P(x))* transforms *a vector N* inputs ($x=(x_{1},....,x_{N})\in R_{+}^{N}$) into a vector of *M* outputs ($y=(y_{1},....,y_{M})\in R_{+}^{M}$). Applying basic economic axioms,^24^ the production set provides a convex and freely disposable input and output technology. Technical efficiency and CU ratio (CUR) of observed input and output (x, y) vectors are derived from the production technology *P(x)*. Output-oriented efficiency scores are estimated by the following output distance function:

(1) $$D_{0}(x,y)=\underset{\theta }{\inf}\{\theta >(y/\theta )\in P(x)\}$$

DEA programs estimate the distance function and CURs.^8,9^ Färe^11,12^ derived CU in a frontier setting using distance function while Ferrier^23^ employs the directional distance function to aggregate capacities of individual hospitals into a group.

We formulate a dual DEA program with shadow prices constraints using the output distance. For each hospital, the dual DEA estimates CU using individual hospital observed inputs and outputs according to Johansen’s definition. In the short-run, inputs need to be categorized as fixed (*x*^f^) and variable (*x*^v^); that is, *x* = (*x*^f^*, x*^v^*)* for each hospital k. We define *N*^f^ fixed inputs and *N*^v^ variable inputs such that *N*^f^+*N*^v^ = *N*. The fixed inputs sub-vector (*x*^f^) refers to the existing plant and equipment. The activity vector Z is a weight assigned to the observed k^th^ hospital in the linear convex technology combination. Suppose *K* (*k* = 1,…, *K)* hospitals in the data sample. Under variable returns to scale and strong disposability, the production set *P(x)* with fixed and variable inputs is:

(2) $$P(x)=\left\{y|\begin{array}{c} \sum_{k=1}^{K}z_{k}y_{km}\underline{\geq }y_{m},m=1,...,M,\sum_{k=1}^{K}z_{k}x_{kf}\leq x_{f},f=1,...,N_{f,}\\ \sum_{k=1}^{K}z_{k}x_{kv}\leq x_{v},v=1,...,N_{v},\sum_{k=1}^{K}z_{k}=1,z_{k}>0,\forall k=1,....,K \end{array}\right\}$$

Output-oriented efficiency can be provided by the output distance function giving radial proportion scaled-up hospital output projections on the frontier.^11,12^ The value of the output distance function for the *i*^th^ hospital is calculated using dual linear programming.

From a practical point of view, the above radial efficiency measure *D*_o_* (x*^f^*, x*^v^*, y)* is computed relative to the previously defined reference technology *P(x)* by solving a linear program for each observation. This yields a primal DEA linear program. The dual DEA program is derived with shadow price constraints estimating the distance function *D*_o_* (x*^f^*, x*^v^*, y)*. The value of the output distance function for the k^th^ hospital is found by solving the following dual linear programming input and output shadow prices constraints problem (3).

**Figure:**
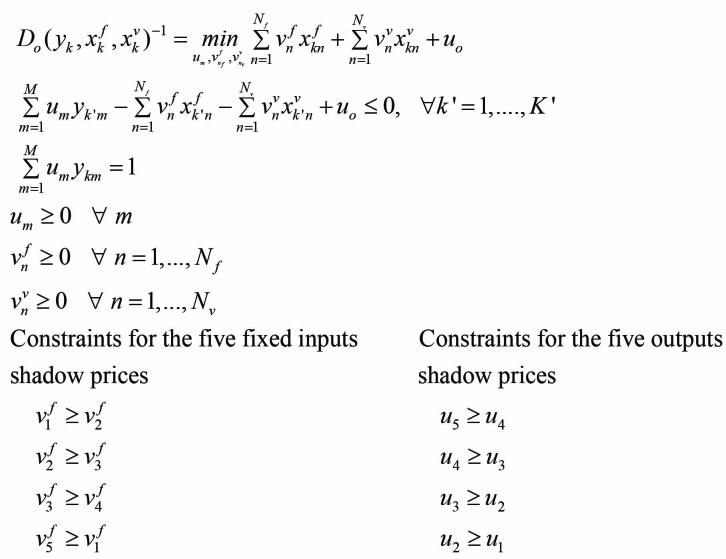

(3)

where

$v_{1}^{f}$ is the shadow price of medical doctors

$v_{2}^{f}$ is the shadow price of surgical dentists

$v_{3}^{f}$ is the shadow price of midwives

$v_{4}^{f}$ is the shadow price of nurses

$v_{5}^{f}$ is the shadow price of beds

u_1_ is the shadow price of outpatients visits in stomatology

u_2_ is the shadow price of outpatients visits in emergency

u_3_ is the shadow price of outpatients visits in external wards

u_4_ is the shadow price of admissions in maternity wards

u_5_ is the shadow price of admissions

Fixed factor shadow prices restrictions impose an input values ordering. Fixed inputs are the number of medical doctors, surgical dentists, midwives, nurses, and beds in the empirical study. Assuming a higher shadow price of one physician compared to one surgical dentist, which is higher than the one of a midwife which is higher than the one of a nurse. The shadow price of a bed (proxy of capital) is assumed higher than those of all other inputs. Following the same line of thought, a shadow prices ordering is imposed among outputs (admissions, admissions in maternity wards, outpatient visits in stomatology, in emergency and in external wards). Admissions are higher valued than outpatient visits. These are expected relationships in the real world. It avoids DEA models to choose inappropriate shadow prices for the evaluated hospitals. That is why it is so important to complement DEA models with sound exogenous economic information.

The second step in measuring the CUR is to determine each hospital’s capacity with constant fixed inputs, allowing variable inputs to be unrestricted (consistent with Johansen’s definition of capacity). Hospital k’s capacity is given by the following linear programming solution (4):

**Figure:**
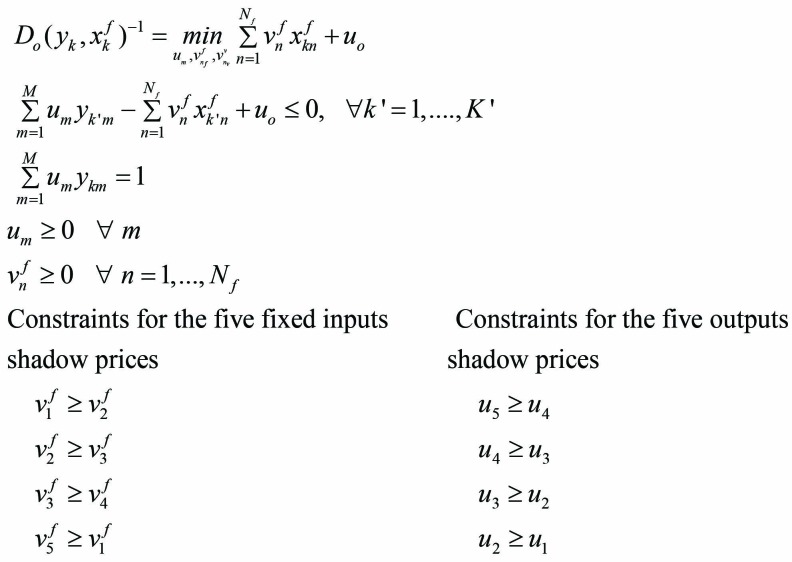

(4)

The only difference between the equations (3) and (4) is the treatment of variable inputs. In equation (3) hospital variable inputs are restricted to currently available levels; in equation (4) variable inputs are unrestricted and not constrained. It is assumed that a hospital has access to as many variable inputs as needed for full capacity. Variable inputs can be omitted from the specification. D_0_(y_k_,x^f^_k_,x^v^_k_)^-1^ and D_0_(y_k_,x^f^_k_) are the inverse of Shephard distance function^18^ and can be interpreted as Farrell’s efficiency measure.^22^ They represent the possible radial increase in outputs if hospital (k) operates efficiently. The last step in the CUR process is taking the ratio of the inverse of solutions given by equations (3) and (4) to determine hospital k’s CUR:

(5) $$CUR(y_{k},x_{k}^{f},x_{k}^{v})=\frac{D_{0}(y_{k},x_{k}^{f})}{D_{0}({\mathrm{(y}}_{k},x_{k}^{f},x_{k}^{v})}$$

This measure is devoid of any technical inefficiency since the latter appears in the numerator and denominator.$1-CUR(y_{k},x_{k}^{f},x_{k}^{v})$ can be interpreted as the percentage of additional output to be produced at full capacity without variable input restrictions. Obviously, given the property of the output distance function necessarily:

$$0<CUR(y_{k},x_{k}^{f},x_{k}^{v})\leq 1$$

Using CUR has several advantages. First, by taking the ratio of the output distance functions any technical inefficiency is removed by definition. This means the measure is not downward biased, in contrast to most traditional CU measures. Second, capacity measures are computed at the frontier and defined relative to observed best practices. Third, it is able to accommodate a multiplicity of inputs and outputs. Input and output data describe the hospital’s production technology without the use of functional forms. The advantage of using a non-parametric approach - not requiring an explicit representation of the form of the technology - is that it envelopes the observed input and output data by forming facets around the frontier of the observed data. This type of modeling has been used extensively in hospital studies as well as in health economics more generally (see Hollingsworth^25,26^ for a review of efficiency measurement and Ferrier^23^ and Färe^11^ for the applied nonparametric measure of CU for health facilities).

### Data

Public hospitals in Tunisia are regionally-based of PDH as a first reference level receiving patients that go beyond the primary healthcare centers. These hospitals regulate access to referral regional and university hospitals with a mission to provide healthcare services for the whole territory. They consume a high proportion of the overall MoH budget (25% in the 2013 budget year). In addition, these hospitals account for about 66% of total public hospitals.

PDH were created the decade following the independence in 1956. It focused on building health infrastructure, when medical treatment uses less specialized technology for high prevalence of communicable diseases. More PDH were created in the 1980s to support primary healthcare, especially maternal and child health. Their mission as defined by the sanitary law 1991 was limited to provide ambulatory and emergency care, maternal and child care and short day’s hospitalizations. PDH offer many healthcare services such as outpatient, inpatient and emergency of internal medicine, surgery, as well as pediatric, dental, and maternity care. Outpatient and inpatient care are the first services delivered while the second set essentially consists of emergency. Medical consultations of the PDH represent more than 40% of total medical visits of all public health facilities. Overall, the volume of medical services increases over the period.

The size of the PDH is relatively small with on average of 38 beds and 16 physicians. Large disparities can be detected by the variation in the number of beds ranging from 20 to 128 and in the number of physician ranging from 8 to 26 (Table 1), Overall, the size of PDH, as measured by human and physical resources, increased over the study period. Indeed, of the 166 of total public’s hospitals in the country, 105 are PDH with 15% of total public beds in 2010 vs. 12% in 2000.^2,3^ PDH are managed and subsidized MoH. One of the most striking observations is that social health insurance contributes indirectly to the finance of PDH; contrary to the billing system implemented for the regional and university hospitals.

Relevant data of the PDH is limited in Tunisia. In this study, data was collected from various MoH reports for 2010^2^ and from a survey for 2000.^27^ The CU is estimated for 101 of 105 PDH in 2000 and 94 of 105 PDH in 2010.

Inputs and outputs were selected according to the literature on nonparametric DEA-based measurement of hospital efficiency.^25,26,28,29^ PDH production technology was represented by five fixed inputs, one variable input and five outputs, common to all hospitals. The input variables are broadly classified into labor, capital and technological input. The input variable of ‘staff’ consists of total staff of a particular hospital. The break-down of the total staff in terms of the number of doctors, number of surgical dentist, number of midwives and number of nurses was available.

Two measures of the capital input were used, one based on the number of beds per hospital as proxy for net capital assets (see Färe^11^) and one being the operating budget. The operating budget includes expenditure on drugs, maintenance of medical equipment, machinery, vehicles, infrastructure, etc as a proxy of the quantity of capital investment.

In general, hospitals provide three major services: outpatient, inpatient, and laboratory services. Ideally, health output should be measured as an increment to patient health status as final products of hospitals. However, since this is technically impossible to measure, in all hospital CU and efficiency studies intermediate outputs of various kinds are used instead. Outputs disaggregated into inpatient and outpatient output is used in many studies.^30,31^ Given the complex definition of hospital output due to limited data availability in PDH, the number of cases treated in outpatient and inpatient services handled in five categories was chosen as a representative measure of the hospital’s output, since these were assumed to have significant implications for the use of resources. The selected outputs are services that supposedly improve health status. Descriptive statistics of input and output variables are provided in Table 1.

**Table 1:** Descriptive Statistics of Input and Output Variables, 2000 and 2010

	**2000**				**2010**			
	**Mean**	**SD**	**Min**	**Max**	**Mean**	**SD**	**Min**	**Max**
			**Inputs**
No. of physicians	7.62	3.83	6.00	22.00	16.01	2.82	8.00	26.00
No. of surgical dentist	1.11	0.62	0.00	3.00	4.20	1.20	0.00	9.00
No. of midwives	6.03	2.33	0.00	17.00	10.42	3.22	0.00	14.00
No. of nurses and equivalents	40.72	19.81	12.00	94.00	88.38	16.23	46.00	116.00
No. of beds	24.08	19.14	15.00	84.00	38.12	24.41	20.00	128.00
Operating budget	305.10	152.00	100.10	441.00	484.80	78.10	250.80	1260.00
			**Outputs**
Outpatient visits in stomatology ward	3155.07	2056.21	0.00	11274.00	5626.83	18965.42	0.00	32145.00
Outpatient visits in emergency ward	10533.26	8221.50	0.00	43389.00	22134.51	9001.50	0.00	76250.00
Outpatient visits in external wards	27146.71	18809.64	0.00	108378.00	36445.12	14320.00	560.00	322446.00
No. of admissions	518.92	586.82	0.00	3191.00	1424.67	724.41	450.00	10320.00
No. admissions in maternity wards	441.82	309.21	0.00	1512.00	764.63	816.82	0.00	4316.00

Abbreviation: SD, standard deviation‏.

The availability of data on various indicators in the PDH was limited in Tunisia and, due to this constraint, we had to restrict our analysis to the above-mentioned input and output variables. Even we could not get data for the selected variables for 4 PDH in 2000 and for 11 in 2010; therefore, we could only include the remaining PDH out of 105 in our study. The data included in the study were reliable as we conducted checks and found that they were of good quality.

A DEA model for the measurement of CUR was run after feeding the input and output variables into program equation 5. The CUR has economic relevance in the short term. The physical inputs are fixed (physicians, surgical dentists, midwives, nurses, and beds). Considering the operating budget as the only variable input, 1 minus CUR can be interpreted as the additional output if the budget were increased given the existing fixed inputs. In the short term, a low CUR is due to an insufficient budget. In the long run, the size (number of beds) and personnel categories can be considered as variable too.

## Results

Table 2 summarizes the CUR results for the two years according to the standard DEA (CUR_SDEA_) and dual DEA model with shadow prices restrictions (CUR_PDEA_). Overall, PDHs are not operating at full CU with respect to the two DEA models.

**Table 2:** Descriptive Statistics of CUR 2000-2010

		**No. of PDH**	**Mean**	**SD**	**Min**	**Max**
CUR_SDEA_	2000	101	0.95	0.07	0.60	1.00
	2010	94	0.92	0.11	0.66	1.00
CUR_PDEA_	2000	101	0.87	0.15	0.22	1.00
	2010	94	0.82	0.13	0.41	1.00
**Wilcoxon Test**						
CUR_SDEA_ vs. CUR_PDEA_ 2010	Observed statistics = 5518		*P* value =.042			
CUR_PDEA_ 2000 vs. CUR_PDEA_ 2010	Observed statistics = 5198		*P* value =.055			

Abbreviation: CUR‏, capacity utilization ratio.

The average scores of CUR_SDEA_ decreased from 0.95 in 2000 to 0.92 in 2010, indicating an excess capacity of 5% in 2000 and 8% in 2010. However, the average scores of CUR_PDEA_ decreased from 0.87 to 0.82, yielding to excess capacity in PDH of 13% in 2000 and 18% in 2010. Given their fixed resources, PDHs capacity usage could be increased by around 13% in 2000 and 18% in 2010 even under a budget constraint. PDHs had highest average CU in 2000 compared to 2010 meaning that the excess capacity of PDHs is less in 2000 than in 2010. The PDHs in 2010 are more likely operating farther from full plant capacity.

Based on the standard DEA model, the CUR_SDEA_ for PDHs varies from 0.60 to 1 in 2000 and from 0.66 to 1 in 2010. CUR_PDEA_ varied from 0.22 to 1 in 2000 and from 0.41 to 1 in 2010 and in average decreased by 5% when comparing 2010 to 2000. It is clear that the CUR measured by dual DEA model is less than the standard one. This is not surprising because the dual model includes a larger set of restrictions on input and output variables. Thus, we can infer that our Dual DEA-based shadow price results are quite robust. In the rest of the analysis, we focus on the trends in CUR_PDEA_ measures.

It is very difficult to see whether the standard du dual DEA models have significant differences just by comparing some statistical descriptions. A parametric test would be based on the framework of Simar and Wilson^32,33^ that developed a statistical framework for DEA to estimate confidence intervals of efficiency scores. However, their method relies on a standard DEA output or input model for which the data generating process (DGP) can be rigorously formulated from a statistical point of view. In our case, we use a non-standard DEA model in the sense that the CU measure is a ratio of two efficiency measures and, in addition, we use a dual model including ordering on the shadow prices. Therefore, the framework of Simar and Wilson cannot be applied in our study. There is no statistical model in the literature available for CU measure. An alternative is to use nonparametric Wilcoxon test for unknown distribution and independent samples like the case of the distribution of CU scores. Thus, we used Wilcoxon to test the differences DEA models for 2010. We find evidence of no significant differences between CU scores of 2010 between standard and dual DEA models (*P *= .042). The same test rejects the statistically significant differences of CU dual DEA models for the two study periods (*P *= .055).

Figure gives the frequency distribution of CUR_PDEA_ and classifies the hospitals by their CU scores. These results indicate that 76% of PDHs operate under their full plant capacity in 2010, whereas in 2000 only 73% of PDHs operate in the same condition. Sixty-five percent of total PDHs use more than 80% of their capacity in 2010 whereas in 2000 they use only 72%. The standard deviation of CUR_PDEA_ is 0.15 and 0.13, respectively, denoting a quite homogenous frequency distribution.

**Figure:**
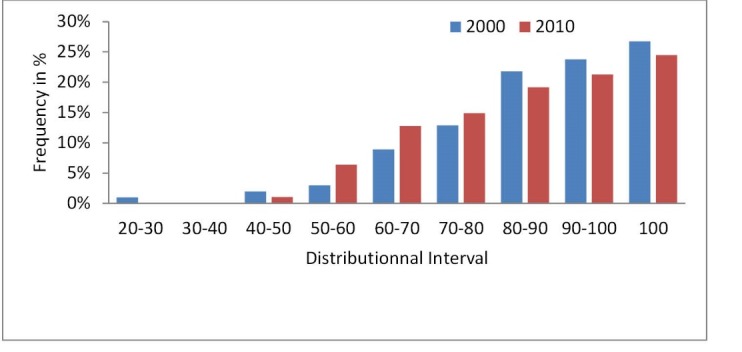
Frequency Distribution of Plant Capacity Utilization (CU).

Table 3 presents the frequency distribution of the utilization of the variable input (operating budget) (VIU). VIU distribution is similar to that obtained for CUR_PDEA_. The PDHs can be classified as follows:

**Table 3:** Frequency Distribution of VIU 2000-2010

	2000	2010
VIU <1	73	65
VIU = 1	7	5
VIU >1	21	24

Abbreviation: VIU, variable input.

- Group I (73 PDH in 2000 vs. 65 in 2010) has a VIU <1 meaning having an excess budget with respect to the optimal CU.
- Group II (7 PDH in 2000 vs. 5 in 2010), has a VIU = 1 meaning optimal budget use.
- Group III (21 PDH in 2000 vs. 24 in 2010), has a VIU>1 meaning needing more budget.

Moreover, we establish a comparison between CUR and the classical BOR. Table 4 shows that both indicators are completely different and not correlated. On average, BOR shows that only 34% (2000) and 38% (2010) of beds are used while CUR shows, respectively, 87% and 82% of the plant capacity is used. The correlation-coefficient is barely 8% in 2000 and 12% in 2010, meaning a full CU of hospitals does not imply efficiency in bed occupation.

**Table 4:** Correlation CUR and BOR, 2000 and 2010

	**2000**		**2010**	
	**CUR**_PDEA_	**BOR**	**CUR**_PDEA_	**BOR**
Average	0.87 (0.15)	0.34 (0.18)	0.82(0.13)	38 (0.20)
No. of PDH	101		94	
Correlation	0.08		0.12	

Abbreviations: CUR‏, capacity utilization ratio; BOR, bed occupancy rate‏.

The BOR is measuring utilization of the available bed capacity expressed as a percentage of occupied beds in a defined period of time. BOR is an output indicator measuring a single dimension while CU includes the multiproduct nature of the hospital production. A low BOR can be due to limited staff numbers. A low CU can be improved by a more flexible budget to fully exploit the fixed inputs, in short term.

The national development policy framework in Tunisia is based on five-year plan that broadly comprises socio‐economic areas. In order to, optimally manage the economic development across the region, policy-makers divided Tunisia in seven major economic regions and established three offices Regional Development in 1994. The purpose of these measures is to include gradually the trends in regional development policy into the five-year development plans. Then, we study the regional distribution of the PDHs. By region, PDHs may have different technical capacities, which can reflect differences in wealth of catchment areas. Table 5 shows that PDHs in the great Tunis area have the highest average CU meaning they are near the full plant CU given their fixed inputs. On the opposite, PDHs located at the South Western region display the lower CU, meaning that they could increase their services by 23% in 2010 given the current fixed inputs and an unconstrained operating budget. By the Kruskal-Wallis test of the CUR_PDEA_ per region for the two years (Table 5), we can claim that CU of PDHs is not significantly different.

**Table 5:** Distribution of CUR_PDEA_ per Region

	**2000**		**2010**	
	**No. of PDH**	**Mean**	**No. of PDH**	**Mean**
Great Tunis	2	0.97 (0.03)	3	0.94 (0.01)
North East	12	0.91 (0.10)	9	0.87 (0.05)
Western North	18	0.85 (0.13)	18	0.80 (0.08)
Centre Western	24	0.89 (0.12)	20	0.84 (0.08)
Centre East	24	0.87 (0.16)	23	0.81 (0.11)
South East	9	0.93 (0.05)	10	0.86 (0.10)
South Western	12	0.80 (0.21)	11	0.77 (0.18)
*P* values Kruskal-Wallis test for equality of population of CU	.833		.542	

Abbreviations: CUR, capacity utilization ratio; CU, capacity utilization; PDH, Public district hospital.

## Discussion

The present study evaluates the CU of PDH using the standard and the dual DEA models. The model that we developed is a dual form of the standard DEA where we integrate restrictions on shadow prices of outputs and inputs. The new version of the DEA model provides a better and more robust economic interpretation of the production frontier and in particular the nonparametric measure of the CU.

The CU scores reported in this paper are based on hospital inputs and outputs data for 2000 and 2010. We found that CU scores have decreased from 2000 to 2010. Our results shown in Table 2 suggest that while it is effectively true that the hospital have become more equipped in 2010, their budget has not followed this tendency and, therefore, their CU was decreased. These results are in the same range as those obtained by Ferrier^35^ and Karagiannis^36^; 87% of US hospitals and 15% of Greek hospitals did not operate at full capacity, respectively. However, the Valdmanis^37^ study of plant capacity shows that public hospitals in Thailand are generally operating at relatively high capacity (90%–95%), given the levels of fixed inputs. We cannot compare these scores to our results, as they do not specify similar technology and dual DEA model with shadow prices restrictions.

The dual DEA models easily include multi-output and multi-input factors of hospital production and measure CU more accurate than the often used BOR. We found a wide variation in the capacity DEA-based measure and BOR (0.34 versus 0.87 in 2000 and 0.38 versus 0.82 in 2010). VIU information - defined as optimal use of the operating budget over actual usage - was used as a recommendation. A CU of 0.80 indicates that full capacity can be obtained if the operating budget is increased by a 20%.

There is no correlation between CUR and BOR. Zere^34^ has found similar results and concludes that a CU-based DEA measure is more suitable for multiproduct hospitals. BOR is considered a doubtful and inappropriate indicator to evaluate the capacity of hospitals. The dual DEA-based CU method gives rich diagnostic information offering causes and recommendations on erasing excess capacity. The low rates (of CU and BOR) may imply that patients are not utilizing these hospitals and bypass their services to use second and third levels of public health facilities or to use private facilities. The poor quality, the lack of a diversification of specialized services as well as the lack of specialized physicians are part of the cause of the low CU.

In the short term, the increase of the budget is a crucial solution for these hospitals and it can be increased by the SHI payments and/or the state subsidies. In fact, much has happened over the period in terms of health sector development and the reform of SHI. The new designed SHI, as purchaser of health services, has favored the coverage of healthcare services delivered at regional and university hospitals as well as private facilities, ignoring the PDHs and basic healthcare centers. It is crucial that SHI reinforces the PDHs supply to better satisfy the users and consequently reduces bypassing. Other solutions would be to reduce copayment for some services for patients and make some services such as child and motherhood care completely free of copayments in order to increase the use of PDH services.

Our study lacked a complete overview of all information related to demand side (such as the perception of the lack of quality, unmet needs, and the level of OOP) or supply side factors (such as lack adequate specialized physicians, as well as equipment and other medical goods), as many of them are unmeasured. However, both demand and supply side are important. The influence of demand and supply side separately on CU is important and needs to be further investigated. A crucial question in this discussion is whether the hospitals could afford to provide expanded services and whether these could be provided with high quality.

Epidemiological and demographic transitions as well as the intense use of technological improvements in referral hospitals (regional and university) have contributed to lower use of PDH. This transition in Tunisia is characterized by decreasing rates of infectious diseases and increasing rates of cardiovascular, respiratory, and neurodegenerative diseases. With such changing disease profile, PDHs as the first line of the public hospitals network, has not accordingly adapted. Chronic disease management indicates that high percentages of patients directly seek treatment in referral hospitals. The PDHs requirements for chronic disease treatment are likely to be low. Due to changes in medical technology and the sub-optimal responsiveness to community expectations, outpatient and inpatient services are done in second and third levels.

In Tunisia, all public hospitals fall under central level MoH management deciding on recruitment and allocation of resources. Current budget allocation seems insufficient to guarantee that all PDH are producing at full capacity. Expanding PDH budget is possible by increasing revenues (via household’s direct payments and/or the financial support of the social health insurance funds) or increasing state subsidies. Inflexibility in reallocation and historic budget policy are the key issue in solving this problem.

The analysis of CU per region highlights three points. First, the CU is reduced by more than 4% in Western North, Centre Western, Centre East and South East between 2000 and 2010. Second, Great Tunis always registered the highest average score of CU whatever the considered year. Compared to others regions, PDHs of Great Tunis has the lower unused capacity indicating that the volume of output can be increased by only 3% in 2000 and 6% in 2010, with respectively, the actual fixed quantity of inputs and an unconstrained operating budget. Third, South Western is on the opposite, ranked among the regions recording the highest average unused capacity level (23% in 2010). CU variations between regions can be partly explained by ability to obtain direct household revenues. Increasing the operating budget seems a key solution for better utilization of fixed inputs. PDH able to attract additional finance are favored. From an equity point of view, state subsidies favoring Center and South Western regions can probably help CU increase.

PDHs are unable to provide a complete needed treatment due to the lack of specialized physicians and medical equipment and technology. For example, women seeking safe, acceptable and good quality maternal and childbirth health services prefer the better-functioning and better equipped regional and university hospitals. PDHs are facing structural and managerial problems related to human resources (particularly the severe shortage of specialized physicians), machinery and operating rooms. They need to constitute an effective continuum of both preventive and curative services for most of the common diseases and to facilitate smoother functioning of primary healthcare centers.

The majority of PDHs is facing limited resources in dealing with the unfavorable socio-economic circumstances and underserved catchment area. They do not provide the services requested. Improving the management of the PDHs requires the coordinated deployment of resources to better serve the population. Under the decentralization plan some PDHs have to be upgraded as regional hospitals to ensure an increased CU in the underserved area.

Urgent steps need to be taken to enlarge the CU of PDH. Government should provide better working and living conditions in the peripheral areas to encourage physicians and other health personnel to work at PDH. In addition, SHI should turn as direct purchaser of curative care from the PDH as well as for a package of preventive services.

Our results should be limited to the short term perspective regarding the composition of the fixed physical inputs. Our findings show convincingly that there is scope to employ CU-based DEA models to manage hospital networks and to reallocate resources at a strategic and operational level. The objective to address allocation decisions is met. For each specific PDH, the DEA model has identified CUs that could be used as comparators. The under-utilization capacity of PDH can learn from their fully CU peers by observing their production and management process. Reallocation of resources (state subsidies) can be done from higher to lower CU PDHs via the operating budget. By region, CUR variation reflects the differences in economic status of catchment areas of PDHs. Hence, reallocation of resources from more affluent areas where patients have a greater ability to pay for hospital services to poorer areas could be considered.

Additionally, the unused capacity of individual PDH can lead to cost savings since our findings shows that additional operating budget (variable inputs) would be needed to increase the CUR for some hospitals but not for others; in the short-run hospital planning. Furthermore, variation of personnel or beds in the medium and long term, respectively, could lead to others cost saving adjustments. More precisely, the CU based-DEA models can help decision-making in setting priorities and cost saving in terms of infrastructure or human resources and in hospital funding. Therefore, in-depth data and research is needed to reach more detailed hospital CUs geared towards a more complete set of managerial needs and perspectives and, thus to address allocation decisions.

## Limitation of the Study

The dual DEA model has its limitations. First, the DEA-based CU exhibits rich diagnostic information to determine causes of low CU and how to deal with it. We did not have complete and reliable data that could be used to unpack the influence of contextual factors on CU using a second-stage regression analysis. However, this study was a first pilot and dealing with the model only. The model needs to be reproduced and may be applied to other areas of healthcare provision to validate it.

Second, the study’ contribution could be improved if panel data for a sufficiently longer period are available to observe the changes in CU. The data used provide average rates of CUR for a specific year but the CU can vary a lot over the year. Furthermore, the data do not allow DRG or case-mix adjustments for inpatient admissions and outpatient visits. Consequently, different patient types are considered as equals in a productive sense.

## Conclusion

Our data analyses indicate that overall, PDH were under-utilizing the production capacity for the two years of the study. The unused capacity (or excess capacity) is estimated to reach 5% in 2000 and 8% in 2010 when applying the standard DEA while it reaches 13% in 2000 and 18 % based on our dual DEA model.

The distribution of CU over the two periods shows a large increase of the proportion of unused capacity. In both years, PDH can increase their production by 20% or more given the fixed inputs and the unconstrained budget. Moreover, the data show that on average, the proportion of PDH unused capacity has increased in between the two years.

The study presents a less gloomy picture of PDH CU compared to BOR. There is a need for new policy for PDH. PDH should operate efficiently at near full capacity level without facing the lack of sufficient budget and supply. Efforts should be made to enhance the productivity and healthcare quality as well as to reduce patients by-passing these hospitals. SHI can play a crucial role to promote the use of all PDH.

From a methodological point, this study has two main contributions. The first one is that the proposal of CU dual non-parametric model using directional output distance functions is to be preferred in performance measurement. The second one is that dual DEA with shadow prices restriction can be used for computing PDH. Our findings can serve as a resource for Tunisian hospitals planners to administer the CUR when addressing allocation decisions of currently available resources for capacity expansion. They may become aware of the advantages of using DEA methods as a measure of CU.

## Ethical issues

We do not need institutional approval since our study uses published data in MoH’ reports. In the findings, we do not present any names of hospitals or regions. We have also, shared the results of our study in national and international workshops.

## Competing interests

Authors declare that they have no competing interests.

## Authors’ contributions

Design and methodology: CA, HL, and MG; Discussion and policy implications: CA and CVM. All authors revised, read, and approved the final manuscript.

## Authors’ affiliations

^1^Iational Institute of Labour and Social Studies (INTES), University of Carthage, Tunisia, Tunis. ^2^LEM-CNRS, IÉSEG School of Management, Lille, France. ^3^LEFA-IHEC, University of Carthage, Tunisia, Tunis. ^4^Health Economist Expert (free lance).

## Key messages

**Implications for policy makers**

- The underutilized hospital’s capacity is detrimental to good practice in spending and should be regularly measured, analyzed, and prevented.
- The inadequate supply due to the lack of operating budget, of specialized physicians, of bed’s flexibility and of health quality should be tackled to reduce unmet user’s needs and the bypassing of the Public district hospitals (PDHs) and, thus to increase their capacity utilization (CU).
- Social health insurance should be turned into a direct purchaser of curative care from the PDH as well as for a package of preventive services. In line, many PDHs may be transformed into regional hospitals.

**Implications for public**

Government efforts in reducing the unused capacity utilization (CU) and in improving the management of district hospitals could increase the patient use of primary healthcare services and reduce the bypassing of these facilities. Public district hospitals (PDHs) should constitute an effective continuum of both preventive and curative services and facilitate smoother functioning of primary healthcare centers.
